# Clinical Characteristics of Patients with Neovascular Age-Related Macular Degeneration and Responses to Anti-VEGF Therapy: Four-Group Stratification Based on Drusen and Punctate Hyperfluorescence

**DOI:** 10.3390/jcm14238593

**Published:** 2025-12-04

**Authors:** Hiroyuki Kamao, Katsutoshi Goto, Kenichi Mizukawa, Ryutaro Hiraki, Atsushi Miki, Shuhei Kimura

**Affiliations:** 1 Department of Ophthalmology, Kawasaki Medical School, 577 Matsushima, Kurashiki 701-0114, Japan; k_goto@med.kawasaki-m.ac.jp (K.G.); hiraki@med.kawasaki-m.ac.jp (R.H.); amiki@med.kawasaki-m.ac.jp (A.M.); kimuras@med.kawasaki-m.ac.jp (S.K.); 2 Shirai Eye Hospital, 1339 Takasecho Kamitakase, Mitoyo 767-0001, Japan; mizu-p@shirai-hosp.or.jp

**Keywords:** neovascular age-related macular degeneration, drusen, pachychoroid, punctate hyperfluorescence, aflibercept

## Abstract

**Background/Objectives**: Different disease subtypes in neovascular age-related macular degeneration (nAMD) influence treatment burden, yet existing classifications such as the pachychoroid neovasculopathy (PNV)/non-PNV dichotomy may not fully capture clinical heterogeneity. This study aimed to compare the 12-month outcomes of intravitreal aflibercept (IVA) in treatment-naïve patients with unilateral nAMD stratified by the presence or absence of drusen and punctate hyperfluorescence (PH). **Methods**: This retrospective study included 130 eyes of 130 patients categorized into the Drusen−/PH−, Drusen+/PH−, Drusen−/PH+, and Drusen+/PH+ groups. Their best-corrected visual acuity, retinal thickness, choroidal thickness, number of injections, no-retinal fluid rate during the loading dose regimen, and 12-month retreatment rate following treatment initiation were determined. The primary outcome was 12-month retreatment rate for the four groups, which was determined using Kaplan–Meier curves and log-rank tests. Exploratory metric multidimensional scaling (MDS) was used to visualize the baseline profiles. **Results**: The 12-month retreatment rates of the groups were significantly different. The Drusen+/PH− group had a higher retreatment rate and required more injections than the Drusen−/PH+ and Drusen+/PH+ groups. The Drusen+/PH− group was older than the Drusen−/PH+ and Drusen−/PH− groups. The Drusen−/PH+ group had a thicker choroid than the Drusen+/PH− group. The MDS results clear separation of the groups, consistent with the older age of the Drusen+/PH− group and the thicker choroid of the Drusen−/PH+ group. **Conclusions**: nAMD stratified based on drusen and PH differed in age, choroidal thickness, and IVA outcomes. The four-category framework provides greater pathophysiologic and therapeutic resolution than the simple PNV/non-PNV dichotomy and may help anticipate injection demand to guide individualized dosing strategies.

## 1. Introduction

Neovascular age-related macular degeneration (nAMD) is one of the leading causes of blindness in developed countries. However, the visual prognosis of patients with nAMD has improved substantially since the introduction of anti-vascular endothelial growth factor (VEGF) therapy [1]. Nevertheless, undertreatment remains a risk factor for poor visual outcomes. This necessitates ongoing therapy and imposes economic and time burdens on patients. Fixed dosing, pro re nata (PRN), and treat-and-extend (TAE) regimens have been implemented to address this treatment burden. However, treatment responses vary among patients, and the optimal dosing regimen must be tailored to each patient. Therefore, it is important to identify predictive factors for the efficacy of anti-VEGF therapy to optimize the treatment regimen.

Two pathophysiologic subtypes of nAMD have been recognized [2]. The first is macular neovascularization associated with drusen, which are extracellular deposits that accumulate between the retinal pigment epithelium (RPE) and Bruch’s membrane [3]. The second is macular neovascularization associated with the pachychoroid, which is characterized by pathologic dilation of choroidal vessels (pachyvessels) and choroidal vascular hyperpermeability (CVH) [4]. The pachychoroid diseases include pachychoroid pigment epitheliopathy (PPE) [4], central serous chorioretinopathy (CSC) [5], pachychoroid neovasculopathy (PNV) [6], and polypoidal choroidal vasculopathy (PCV) [7]. Several studies have reported that PNV requires fewer anti-VEGF treatments than non-PNV [8,9]. However, a comparative study of PNV and non-PNV identified patients without both drusen and pachychoroid features. This suggests that dichotomous classifications based on drusen and pachychoroid may be insufficient to facilitate an understanding of the pathophysiology of nAMD [10]. Inoda et al. stratified the patients with nAMD into four groups based on the presence or absence of drusen and pachychoroid. They reported group-specific aqueous humor cytokine profiles and suggested distinct etiologies for the groups [11]. However, the anti-VEGF treatment responses of these four phenotypes have not been reported.

Punctate hyperfluorescence (PH) is frequently observed in the central areas of CVH in most eyes with CSC [12] and PCV [13] during the mid-to-late phases of indocyanine green angiography (ICGA). Both CSC and PCV are classified as pachychoroid diseases. The associations between PH and anti-VEGF treatment outcomes have been reported for nAMD. For example, the retreatment rate after the loading dose regimen of intravitreal aflibercept (IVA) was lower for eyes with PH [14], and the no-retinal fluid rate after switching to intravitreal brolucizumab was higher for those with aflibercept-refractory nAMD [15]. These findings align with those of previous reports on the favorable response of PNV to anti-VEGF therapy and suggest that PH is a characteristic pachychoroid finding.

This study aimed at delineating the clinical phenotypes of nAMD and their varied responses to anti-VEGF treatment based on drusen and pachychoroid-related findings (PH). The findings of this study are expected to enhance pathophysiologic understanding and support the development of individualized treatment regimens.

## 2. Materials and Methods

### 2.1. Study Design

We conducted a retrospective observational study at the Kawasaki Medical School. The medical records of the consecutive treatment-naïve patients with unilateral nAMD who underwent IVA at our hospital between January 2016 and December 2020 were reviewed. The demographic data and information on systemic comorbidities, including hypertension, diabetes, and smoking status, were extracted from the hospital records. Smoking status was categorized as never-smoker or ever-smoker based on a previously published classification [16]. The exclusion criteria were as follows: having undergone laser photocoagulation or vitrectomy; having macular neovascularization secondary to high myopia (>−6 diopters), uveitis, or angioid streaks; and having other ocular diseases potentially affecting the outcomes in the study eye, such as branch retinal vein occlusion, diabetic retinopathy, or glaucoma.

### 2.2. Treatment Method and Data Collection

All enrolled patients were administered three consecutive monthly IVA injections as a loading dose. They subsequently underwent monthly monitoring for up to 12 months. The eyes with residual or recurrent retinal fluid detected on optical coherence tomography (OCT) after the loading dose regimen were classified into the retreatment group and treated using the TAE protocol. The retinal fluid was defined as subretinal fluid (SRF) and/or intraretinal fluid (IRF).

**Figure 1:**
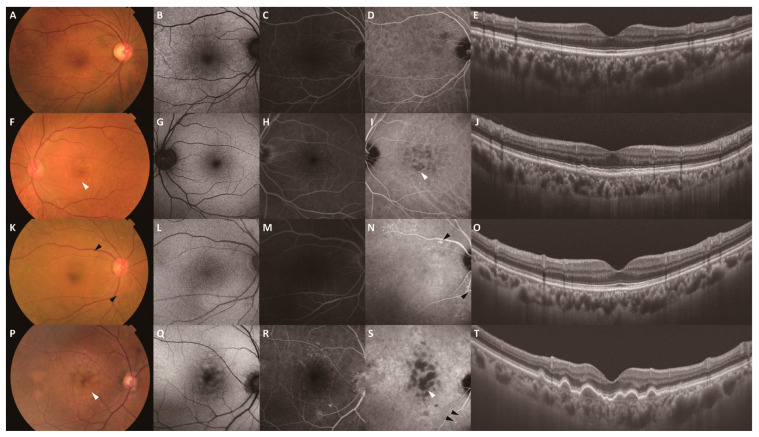
Representative multimodal images of the fellow eyes stratified by drusen and PH. Images (**A**–**E**) show the multimodal images of a 69-year-old woman in the Drusen−/PH− group. Images (**F**–**J**) show the multimodal images of a 72-year-old man in the Drusen+/PH− group. Images (**K**–**O**) show the multimodal images of a 75-year-old woman in the Drusen−/PH+ group. Images (**P**–**T**) show the multimodal images of an 87-year-old man in the Drusen+/PH+ group. All images were obtained at the start of treatment. CFP (**A**), FAF (**B**), late-phase FA (**C**), and late-phase ICGA (**D**) images and a B-scan swept-source OCT image of the macula (**E**) show no remarkable changes. (**F**) CFP showing yellow-white aggregates with poorly defined borders in the macula (white arrowhead). (**G**–**I**) FAF, late-phase FA, and late-phase ICGA images showing the hypofluorescent area corresponding to the yellow-white aggregates (white arrowhead: drusen). (**J**) A B-scan swept-source OCT image of the macula showing pigment epithelial detachment. (**K**) CFP showing scattered yellowish deposits over the vascular arcades (black arrowhead). (**L**–**N**) FAF, late-phase FA, and late-phase ICGA images showing the hyperfluorescent area corresponding to the yellow-white aggregates (black arrowhead: PH). (**O**) A B-scan swept-source OCT image of the macula showing no remarkable changes. (**P**) CFP showing yellow-white aggregates (white arrowhead: drusen) with poorly defined borders in the macula. (**Q**–**S**) FAF, late-phase FA, and late-phase ICGA images showing hyperfluorescence (black arrowhead: PH) over the lower vascular arcade and hypofluorescence corresponding to the yellow-white aggregates in the macula (white arrowhead: drusen). (**T**) A B-scan swept-source OCT image of the macula showing pigment epithelial detachment. CFP, color fundus photography; FAF, fundus autofluorescence; FA, fluorescein angiography; ICGA, indocyanine green angiography.

All participants underwent comprehensive ophthalmologic examinations, including measurement of best-corrected visual acuity, indirect ophthalmoscopy, slit-lamp biomicroscopy with a noncontact lens, color fundus photography (CFP), fundus autofluorescence (FAF) (HRA-2; Heidelberg Engineering GmbH, Dossenheim, Germany), swept-source OCT (DRI OCT-1 Atlantis; Topcon Corporation, Tokyo, Japan), fluorescein angiography (FA), and ICGA (HRA-2; Heidelberg Engineering GmbH, Dossenheim, Germany). Visual acuity was measured in decimal notation and converted to logarithm of the minimum angle of resolution (logMAR) units for analysis. Central retinal thicknesses (CRTs) and subfoveal choroidal thicknesses (SFCTs) were measured using swept-source OCT based on previously published methods [15]. Drusen and PH were assessed in all fellow eyes based on previous studies [13,17]. The drusen subtypes were determined based on the findings of CFP, OCT, and ICGA. Drusen positivity (Drusen+) was based on the presence of soft drusen and/or subretinal drusenoid deposits (SDDs). Soft drusen were defined as yellow-white aggregates on CFP that corresponded to sub-RPE accumulation on OCT. They had sizes of ≥125 µm and round or ovoid shapes, and their borders were poorly defined. They were typically tightly packed or confluent in the macula and associated with hypofluorescence area on late-phase ICGA. SDDs were defined as the presence of ≥10 discrete whitish deposits on CFP that corresponded to subretinal accumulation on OCT. PH was observed in the mid-to-late phases of ICGA. It was typically distributed along the choroidal vessels and appeared as solitary or clustered hyperfluorescent spots within the CVH areas. Two masked retinal specialists (H.K. and K.G.) independently assessed the presence or absence of PH. Discrepancies were adjudicated by a senior grader (K.M.). Subretinal hyperreflective material (SHRM) in all affected eyes, defined as a hyperreflective signal above the RPE on OCT, was classified as exudation, hemorrhage, neovascular tissue, vitelliform, fibrosis, or absent based on FCP, FAF, OCT, OCT angiography, FA, and ICGA findings, as reported previously [13]. None of the eyes in the present study had the vitelliform or fibrosis types. The patients were stratified into drusen-negative/PH-negative (Drusen−/PH−), drusen-positive/PH-negative (Drusen+/PH−), drusen-negative/PH-positive (Drusen−/PH+), and drusen-positive/PH-positive (Drusen+/PH+) groups based on the presence of drusen (soft drusen or SDD) and PH in the fellow eye. These four prespecified categories were used for all analyses. Representative cases are provided in Figure 1.

### 2.3. Outcome Measures

The primary outcome was the 12-month retreatment rate following treatment initiation across the four groups. The secondary outcomes included baseline characteristics (age, sex, prevalence of hypertension or diabetes, and smoking history); distribution of SHRM subtypes; presence of IRF, SRF, and polypoidal lesions; logMAR, CRT, and SFCT values at baseline and 12 months after treatment initiation; number of injections during the 12 months after treatment initiation; and presence or absence of retinal fluid (SRF or IRF) during the loading dose regimen and for 12 months thereafter. We generated a metric multidimensional scaling (MDS) biplot for the four categories based on six baseline features (age, male sex, ever-smoking, hypertension, diabetes, and SFCT). Each feature was z-standardized across the groups, and the group centroids were mapped in two dimensions. The feature directions were overlaid using post hoc vector fitting (envfit). The permutation *p*-values are provided for descriptive purposes and were not used for confirmatory inference.

### 2.4. Statistical Analysis

Statistical analyses were performed using JMP Pro 17 (SAS Institute, Cary, NC, USA). Age was compared across the four groups using the Kruskal–Wallis test. This was followed by pairwise comparisons using the Wilcoxon rank-sum test with Holm correction. Pearson’s chi-squared test was used to assess the differences in sex distribution, prevalence of hypertension or diabetes, smoking history, distribution of SHRM subtypes, and frequencies of IRF, SRF, and polypoidal lesions across the four groups. Linear mixed-effects models were used to analyze the logMAR, CRT, and SFCT values at baseline and 12 months after treatment initiation, and the number of injections administered during the 12 months after treatment initiation. The least-squares means (LS means) were estimated from the mixed models, and the Tukey–Kramer method was used for post hoc group contrasts. The no-retinal fluid rate during the loading dose regimen and the 12-month retreatment rate were determined using Kaplan–Meier analysis and compared using log-rank tests. Cox proportional-hazards models were used to estimate hazard ratios with 95% CIs. The correlations among the variables (age, sex, smoking history, hypertension, diabetes, and SFCT) were assessed using Spearman’s rank correlation to determine the potential impact of multicollinearity on the multivariable analyses. The severity of multicollinearity was assessed by calculating the variance inflation factor (VIF) for each pair of correlated variables. A VIF value of < 5 was considered acceptable, indicating negligible collinearity. Statistical significance was set at *p* < 0.05.

## 3. Results

### 3.1. Clinical Characteristics of the Study Population

The data of 130 eyes of 130 treatment-naïve patients with unilateral nAMD were included. The patients were stratified into the Drusen−/PH−, Drusen+/PH−, Drusen−/PH+, and Drusen+/PH+ groups based on the presence or absence of drusen and PH in the fellow eye. Their baseline characteristics are summarized in Table 1. The age distributions of the groups were significantly different (*p* < 0.001). The Drusen+/PH− group was older than the Drusen−/PH+ (*p* = 0.002) and Drusen−/PH− (*p* = 0.040) groups. The Drusen+/PH+ group was also older than the Drusen−/PH+ group (*p* = 0.040). The other pairwise comparisons were not significant. The prevalence of diabetes also differed among the groups (*p* = 0.02), with the Drusen−/PH− group having the highest prevalence (42.9%). However, the pairwise comparisons revealed no significant differences (all adjusted *p* ≥ 0.11). The presence of polypoidal lesions differed significantly among the groups (*p* = 0.005). The Drusen+/PH− group had a lower prevalence of polypoidal lesions than the Drusen−/PH− (*p* = 0.03) and Drusen−/PH+ (*p* = 0.01) groups; this was evident as a trend on comparing the Drusen+/PH− group with the Drusen+/PH+ group (*p* = 0.058). No other pairwise differences were significant. The groups did not significantly differ in sex distribution, prevalence of hypertension, smoking history, distribution of SHRM subtypes, or presence of IRF and SRF.

### 3.2. Twelve-Month Outcomes of IVA Therapy

**Figure 2:**
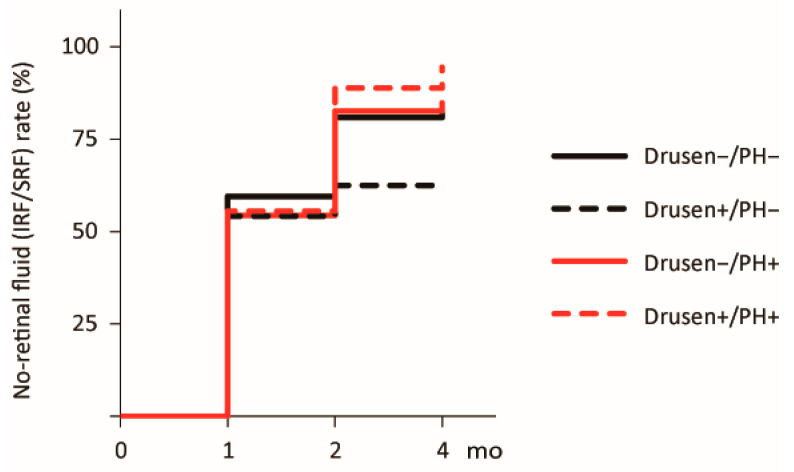
Kaplan–Meier curve for the no-retinal fluid rate following the loading dose regimen. Curves for the times to resolution of retinal fluid (IRF/SRF) after treatment initiation are shown. The black solid, black dashed, red solid, and red dashed lines represent the Drusen−/PH−, Drusen+/PH−, Drusen−/PH+, and Drusen+/PH+ groups, respectively. IRF, intraretinal fluid; PH, punctate hyperfluorescence; SRF, subretinal fluid.

The no-retinal fluid rates at 4 months after treatment initiation were 83.3% (34/42), 62.5% (15/24), 84.8% (38/46), and 94.4% (16/18) for the Drusen−/PH−, Drusen+/PH−, Drusen−/PH+, and Drusen+/PH+ groups, respectively. Kaplan–Meier analysis revealed no significant difference (*p* = 0.26; Figure 2).

**Figure 3:**
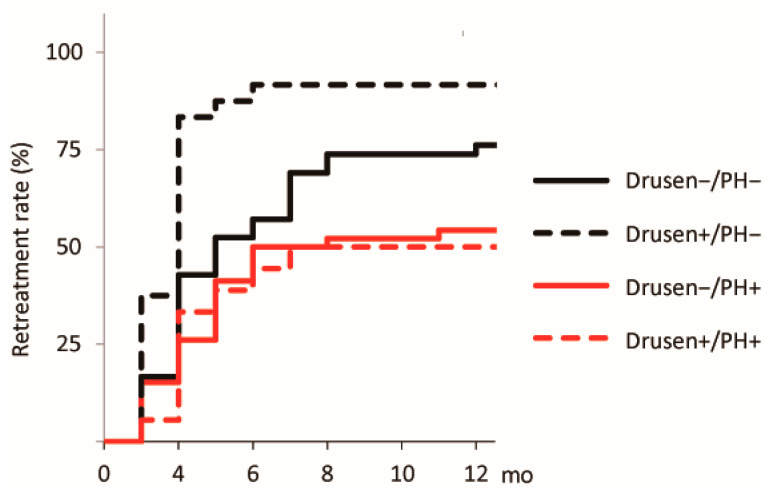
Kaplan–Meier curve for the 12-month retreatment rate after the initial treatment. A curve for the time to retreatment after the initial treatment is also shown. The black solid, black dashed, red solid, and red dashed lines represent the Drusen−/PH−, Drusen+/PH−, Drusen−/PH+, and Drusen+/PH+ groups, respectively. PH, punctate hyperfluorescence.

The treatment outcomes at 12 months after the initial treatment are summarized in Table 2. The logMAR and CRT values did not differ among the groups at baseline and 12 months after the initial treatment. In contrast, the SFCT values differed significantly (F = 7.30 and 6.40, respectively, *p* < 0.001 for both). The choroidal thicknesses of the Drusen−/PH+ group at baseline and 12 months after the initial treatment (282.2 and 248.2 µm, respectively) were significantly greater than those of the Drusen+/PH− group (*p* < 0.001 for both). The other pairwise differences were not significant. The number of injections differed significantly across the groups (F = 4.71, *p* = 0.004). The Drusen+/PH− group required more injections than the Drusen−/PH+ (*p* = 0.002) and Drusen+/PH+ (*p* = 0.046) groups. The other pairwise differences were not significant. The 12-month retreatment rates for the Drusen−/PH−, Drusen+/PH−, Drusen−/PH+, and Drusen+/PH+ groups were 76.2% (32/42), 91.7% (22/24), 54.3% (25/46), and 50.0% (9/18), respectively. The differences were statistically significant (log-rank *p* < 0.001; Figure 3). The Drusen+/PH− group had higher 12-month retreatment rates than the Drusen−/PH− group (hazard ratio [HR] = 2.05; *p* = 0.016; 95% confidence interval [CI]: 1.14–3.67), Drusen−/PH+ (HR = 3.22; *p* < 0.001; 95% CI: 1.70–6.13), and Drusen+/PH+ (HR = 3.54; *p* = 0.002; 95% CI: 1.60–7.84) groups.

### 3.3. MDS Analysis

**Figure 4:**
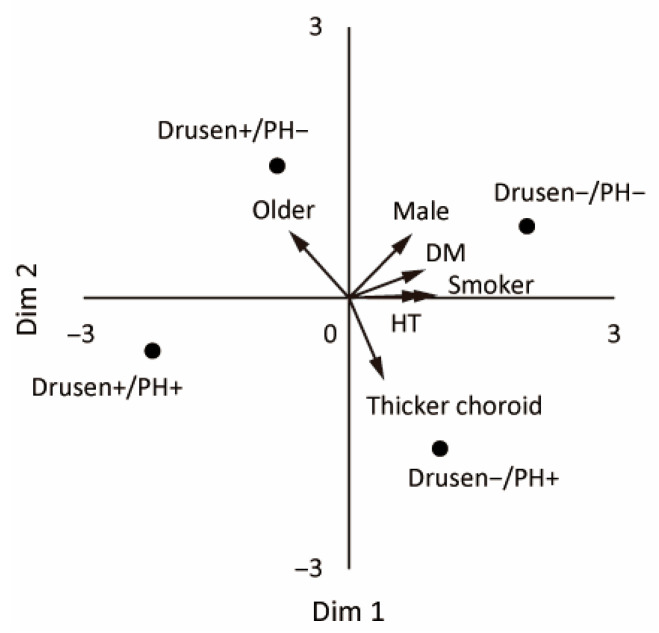
Metric MDS biplot of the four etiology-based subgroups. The points (Drusen−/PH−, Drusen+/PH−, Drusen−/PH+, and Drusen+/PH+) are the group centroids based on the log- and z-standardized baseline features (age, sex, ever-smoker, hypertension, diabetes, and SFCT). The arrows represent the post hoc fitted vectors (higher values in the arrow direction).

We used metric MDS to visualize the etiology-based classification without any assumptions. The group centroids (means of log/z-standardized age, sex, smoking history, hypertension, diabetes, and SFCT) were mapped in 2D, and the four groups were well separated (Figure 4). The biplot indicated positive alignments of Dimension 1 with ever-smoker status, hypertension, diabetes, and male sex (loadings ≈ 0.99, 0.87, 0.86, and 0.70) and a negative alignment with older age (loading ≈ −0.68). Dimension 2 was positively aligned with older age and male sex (loadings ≈ 0.74 and 0.71) and negatively aligned with greater SFCT (loading ≈ −0.90). The Drusen−/PH− group tended to have positive scores for Dimensions 1 and 2. The Drusen+/PH− group had positive displacement along Dimension 2 (older age). The Drusen−/PH+ group had lower scores for Dimension 2 (thicker choroid). The Drusen+/PH+ group was positioned approximately midway between the Drusen−/PH+ and Drusen+/PH− groups when the data were restricted to three categories (excluding the Drusen−/PH− group). This finding is consistent with a mixed phenotype.

Correlation analysis was performed for the six variables (age, sex, smoking history, hypertension, diabetes, and SFCT). Significant associations were identified between smoking status and age (ρ = −0.242, *p* = 0.0055), smoking status and sex (ρ = 0.665, *p* < 0.0001), and SFCT and age (ρ = −0.342, *p* < 0.0001). The VIFs for smoking and age, smoking and sex, and SFCT and age were 1.8, 1.8, and 1.18, respectively. These values are all well below the conventional threshold of 5. These results indicate that some variables were statistically correlated. However, the degree of multicollinearity was minimal, and it was not expected to affect the stability of the regression models.

## 4. Discussion

The treatment-naïve patients with unilateral nAMD were stratified based on the presence or absence of drusen and PH in the fellow eye. This revealed clear heterogeneity in the clinical characteristics and treatment outcomes of IVA. The Drusen+/PH− group consistently had poorer responses to IVA therapy. MDS of the baseline characteristics revealed clear separation among the four groups. The Drusen+/PH− group had a lower prevalence of polypoidal lesions. The Drusen−/PH− group had a higher prevalence of diabetes. The Drusen−/PH+ group had a thicker choroid. These findings indicate distinct clinical profiles. To our knowledge, no prior study has reported the clinical features of the subgroups and their responses to anti-VEGF therapy using a four-category classification using drusen and PH. These findings suggest that the pathophysiology of nAMD differs based on the presence or absence of drusen and PH.

The patients in the Drusen+/PH− group had more injections than those in the Drusen−/PH+ and Drusen+/PH+ groups. Their 12-month retreatment rate was also higher than those of the other three groups. These findings are consistent with those of previous studies that reported more favorable responses to anti-VEGF therapy for PNV (pachychoroid-associated nAMD) than for non-PNV (drusen-associated nAMD) [8,9]. Miyake et al. classified nAMD into PNV and non-PNV and treated the patients with intravitreal ranibizumab under a PRN regimen. They reported no significant difference in the no-retinal fluid rate after the loading dose regimen but a lower retreatment rate after 1 year for the PNV group [8]. Matsumoto et al. classified nAMD into PNV and non-PNV and treated the patients with IVA using a TAE regimen. The number of injections administered over 2 years was significantly lower for those with PNV than for those with non-PNV, with the fewest observed in PNV with polypoidal lesions [9]. The participants in this group were also significantly older, and they were displaced upward along Dimension 2 (reflecting older age) in the MDS plot. Drusen are extracellular deposits that accumulate with age between the RPE and Bruch’s membrane. Their clinical significance as precursors of nAMD and geographic atrophy has been established [3,18]. These findings indicate that this group has drusen-associated nAMD. They also had a lower prevalence of polypoidal lesions. PCV is relatively uncommon in White populations and has historically been considered a subtype of nAMD [19]. It has been recognized as an entity within the pachychoroid disease spectrum following reports of its high prevalence in Asian populations [20] and the establishment of the pachychoroid concept [4]. However, Kuranami et al. reclassified nAMD based on the presence of PNV. The conventional categories (typical AMD, PCV, and retinal angiomatous proliferation) were reclassified into PNV and non-PNV (type 1, 2, and 3 MNV and PCV). They reported that 36.8% of eyes in the non-PNV group had PCV [10]. PCV is diagnosed based on the detection of polypoidal lesions detected via ICGA. Such lesions can develop not only in nAMD but also in radiation retinopathy [21], sickle cell disease [22], and tilted disk syndrome [23]. These findings have fueled debate on whether morphologically defined PCV should be included in the pachychoroid disease spectrum. Our data help reconcile this apparent mismatch. The polypoidal lesions were significantly less prevalent in the Drusen+/PH− group (12.5%). Their prevalence in the Drusen−/PH− (47.6%), Drusen−/PH+ (52.2%), and Drusen+/PH+ (50.0%) groups were comparable. If the categories were collapsed into PNV and non-PNV, the Drusen−/PH− group would be labeled “non-PNV,” which would exaggerate the apparent prevalence of PCV in the non-PNV group. These findings support classifying PCV within the pachychoroid spectrum. They also highlight the value of a four-category framework (Drusen ±/Pachychoroid ±) over a simple dichotomy for interpreting pathophysiology and anticipating treatment needs.

The patients in the Drusen−/PH+ group had a thicker choroid and were clustered lower along MDS Dimension 2 (reflecting thicker choroid). This was consistent with the pachychoroid phenotype. The term “pachychoroid” refers to a disease entity characterized by a thick choroid with dilated choroidal vessels in Haller’s layer and associated attenuation of the choriocapillaris and choroidal vessels in Sattler’s layer [4]. The pathogenesis of pachychoroid diseases is not fully understood. However, they have been hypothesized to involve venous congestion and impaired choroidal outflow, which lead to the development of pachyvessels and CVH and subsequent compression of the choriocapillaris. This compression is believed to cause ischemic damage to the RPE and contribute to disease progression [24]. PH is strongly associated with CVH, which is a characteristic feature of pachychoroid disease. It is also frequently observed in patients with CSC and PCV [12,13]. These observations are consistent with pachychoroid-associated nAMD.

The Drusen+/PH+ group had clinical characteristics and IVA responsiveness comparable to those of the Drusen−/PH+ group (pachychoroid-associated nAMD), except for age. This group occupied an intermediate position between the Drusen+/PH− group (reflecting older age) and the Drusen−/PH+ group (reflecting thicker choroid) on the MDS biplot, which is consistent with a mixed phenotype. Their injection demand and retreatment profiles more closely resembled those of the Drusen−/PH+ group (pachychoroid-associated nAMD) than the Drusen+/PH− group (drusen-associated nAMD), suggesting that they represent older patients with pachychoroid-associated nAMD. These findings suggest that the Drusen−/PH+ and Drusen+/PH+ groups have pachychoroid-associated nAMD, and PH is a key indicator of the pachychoroid phenotype.

The Drusen−/PH− group had a higher prevalence of diabetes. Its MDS position indicated alignment with cardiometabolic covariates, including smoking, hypertension, and diabetes, rather than with drusen-related older age and PH−related thicker choroid. Smoking has been consistently identified as a risk factor for nAMD. A pooled analysis of data from three representative population-based cohort studies (the Beaver Dam Eye Study, the Blue Mountains Eye Study, and the Rotterdam Study) reported odds ratios for the incidence of late AMD for current and former smokers as 2.35 and 1.82, respectively, compared with those for never smokers [25]. Several reports have implicated hypertension as a risk factor for nAMD [26], but two landmark population-based cohort studies (the Beaver Dam Eye Study [27] and the Blue Mountains Eye Study [28]) reported no significant independent association. Some studies have reported associations between diabetes and early AMD [29]. However, diabetes cannot be conclusively regarded to increase the risk of nAMD in the general population. Diabetes is not strongly associated with nAMD. However, the higher prevalence of diabetes in the Drusen−/PH− group suggests that an association in this subgroup cannot be excluded. This group had a more favorable response to anti-VEGF therapy than the Drusen+/PH− group. This pattern suggests proximity to pachychoroid-associated nAMD, which involves choriocapillaris compression and ischemic damage to the RPE. Cardiometabolic covariates may also contribute to choriocapillaris hypoperfusion in the absence of overt pachychoroid. Further targeted study of the Drusen−/PH− subgroup is warranted.

The present study has some limitations. First, the sample size was relatively small. Although baseline clinical characteristics did not differ significantly across groups, this may limit the generalizability of the findings and introduce selection bias. Nevertheless, a post hoc power analysis for the primary endpoint (12-month retreatment) demonstrated adequate statistical power (>90% without and >84% with multiplicity adjustment), supporting the reliability of the main time-to-event comparisons. Second, this study was a retrospective single-center design and consisted solely of Japanese patients. Accordingly, differences across the healthcare system, including variations in the implementation of the TAE regimen and the extension protocol, may influence visual prognosis, anatomical outcomes, or the total number of injections. Even so, several phenotype-anchored features in our four-group classification appear consistent with reported racial tendencies. In the present study, the Drusen+/PH– group, representing the drusen-associated nAMD commonly observed in White populations, showed a lower prevalence of polypoidal lesions. Conversely, the Drusen–/PH+ group, representing the pachychoroid-associated nAMD frequently seen in Asian populations, demonstrated markedly thicker choroids. These findings suggest that, despite differences in the relative frequencies of the four groups among ethnicities, the within-group clinical characteristics are likely to be conserved. Caution is nevertheless warranted when extrapolating treatment effects to populations with different demographic or clinical backgrounds. Prospective, multicenter validation with longer follow-up is warranted. Third, the four-group comparisons of IVA treatment outcomes were conducted as confirmatory statistical analyses based on predefined hypotheses. In contrast, the MDS plot was used solely as an exploratory visualization to illustrate multivariate similarity patterns in baseline characteristics. Because MDS does not support confirmatory statistical inference, its results should be interpreted cautiously and considered descriptive rather than inferential. Although multicollinearity diagnostics indicated acceptable levels, the spatial configuration may still have been influenced by correlated cardiometabolic variables. Therefore, the exploratory findings from MDS require prospective validation in independent, multicenter cohorts. Fourth, drusen and PH were stratified based on the findings of the fellow eyes in this study. However, their identification on ICGA is inherently subjective, and this may have led to misclassification despite the grading being masked. We assessed PH in the fellow eye because hemorrhage and other disease-related changes in the affected eye can mask it and often hinder reliable evaluation. Park et al. reported higher PH detection rates for the fellow eye (PCV, 86.7%; AMD, 60.0%) than for the affected eye (PCV, 86.0%; AMD, 40.0%) in their study of patients with unilateral nAMD [13].

## 5. Conclusions

Stratifying treatment-naïve, unilateral nAMD by the presence of drusen and PH in fellow-eyes delineated clinical characteristics associated with varying responses to IVA. The Drusen+/PH− group had the greatest treatment burden. In contrast, the PH-positive subgroups, regardless of drusen status, had pachychoroid-like profiles and more favorable anti-VEGF treatment outcomes. The low frequency of polypoidal lesions in the Drusen+/PH− group supports the classification of PCV within the pachychoroid spectrum. The four-category framework (Drusen±/PH±) provided greater pathophysiologic and therapeutic resolution than the simple PNV/non-PNV dichotomy and may help anticipate injection demand to guide individualized dosing strategies.

## Figures and Tables

**Table 1:** Clinical characteristics of patients.

Characteristic	Drusen−/PH− (*n* = 42)	Drusen+/PH− (*n* = 24)	Drusen−/PH+ (*n* = 46)	Drusen+/PH+ (*n* = 18)	*p*
Age (years), mean (SD)	72.9 (7.6)	77.8 (8.0)	70.0 (9.0)	75.6 (7.6)	<0.001
Sex (female), no. (%)	8 (19.0)	6 (25.0)	14 (30.4)	6 (33.3)	0.56
Hypertension, no. (%)	23 (54.8)	12 (50.0)	27 (58.7)	5 (27.8)	0.15
Diabetes, no. (%)	18 (42.9)	4 (16.7)	9 (19.6)	2 (11.1)	0.02
Smoking habits (ever-smokers), No. (%)	34 (81.0)	16 (66.7)	35 (76.1)	10 (55.6)	0.20
Presence of polypoidal lesion, No. (%)	20 (47.6)	3 (12.5)	24 (52.2)	9 (50.0)	0.01
Presence of IRF, No. (%)	4 (9.5)	2 (8.3)	5 (10.9)	2 (11.1)	0.99
Presence of SRF, No. (%)	42 (100.0)	24 (100.0)	45 (97.8)	16 (88.9)	0.10
Presence of SHRM, No. (%)					0.67
Exudation	5 (11.9)	4 (16.7)	8 (17.4)	4 (22.2)	
Hemorrhage	10 (23.8)	2 (8.3)	10 (21.7)	3 (16.7)	
Neovascular tissue	1 (2.4)	1 (4.2)	0 (0.0)	0 (0.0)	
No SHRM	26 (61.9)	17 (70.8)	28 (60.9)	10 (55.6)	

IRF, intraretinal fluid; PH, punctate hyperfluorescence; SHRM, subretinal hyperreflective material; SRF, subretinal fluid.

**Table 2:** Twelve-month outcomes of IVA therapy.

Characteristic	Drusen−/PH− (*n* = 42)	Drusen+/PH− (*n* = 24)	Drusen−/PH+ (*n* = 46)	Drusen+/PH+ (*n* = 18)	F, *p*
Baseline, LSMean (95%CI)					
logMAR	0.32 (0.21–0.43)	0.32 (0.18–0.47)	0.20 (0.10–0.31)	0.22 (0.04–0.39)	1.04, 0.38
CRT (μm)	310.0 (277.8–342.1)	305.1 (262.6–347.7)	307.8 (277.1–338.6)	320.9 (271.8–370.0)	0.09, 0.97
SFCT (μm)	230.9 (202.2–259.6)	173.3 (135.3–211.3)	282.2 (254.8–309.6)	230.6 (186.7–274.4)	7.30, <0.001
Month 12, LSMean (95%CI)					
logMAR	0.17 (0.05–0.29)	0.34 (0.18–0.50)	0.11 (−0.01–0.22)	0.07 (−0.11–0.26)	2.33, 0.08
CRT (μm)	216.3 (199.7–232.9)	245.3 (223.4–267.3)	218.5 (202.7–234.4)	231.4 (206.1–256.8)	1.78, 0.15
SFCT (μm)	200.7 (171.2–230.3)	142.8 (103.7–181.9)	248.2 (220.0–276.5)	199.1 (154.0–244.2)	6.40, <0.001
Number of injections, LSMean (95%CI)	5.8 (5.0–6.5)	7.2 (6.2–8.2)	4.9 (4.1–5.6)	5.1 (3.9–6.3)	4.71, 0.004

CI, confidence interval; CRT, central retinal thickness; LSMean, least squares mean; PH, punctate hyperfluorescence; SFCT, subfoveal choroidal thickness. The *p*-values are based on Type III tests of fixed effects (REML, Satterthwaite df).

## Data Availability

The data used to support the findings of this study are restricted by the Kawasaki Medical School Ethics Committee to protect patient privacy. Data are available from Hiroyuki Kamao [hironeri@med.kawasaki-m.ac.jp] for researchers who meet the criteria for access to confidential data.
